# Bishop Temple Reports Progress and Asks for Further Help

**Published:** 1889-05-25

**Authors:** 


### The Bishop of London's Fund.

### BISHOP TEMPLE REPORTS PROGRESS, AND ASKS FOR FURTHER HELP.

On next Sunday sermons will be preached in every church throughout the diocese of London on behalf of the Bishop of London's Fund, which has done so much for Londoners in many ways. In connection with this, and in anticipation of the day, the Bishop of London has issued an interesting pastoral, the substance of which cannot fail to please our readers. Many people will remember the founding of the Bishop of London's Fund, which was the most important event in the history of the year 1864. For years public attention had been gradually drawn to the enormous increase of the population of the metropolis, and to the attendant evils and hardships of the poorer classes. Every year some 50,000 souls were added to the inhabitants of London, but, relatively speaking, nothing was done to increase the agencies which provided for the spiritual and moral welfare of these people. Accordingly, the Bishop of London's Fund was established for supplying the spiritual wants of London and its suburbs. Its objects were to raise contributions to secure the services of missionary clergy labouring in the diocese, but confined in their operations to particular parishes; to build or secure parsonage houses, schools, mission buildings, and churches; to assist towards the endowment in special cases; to further other objects for strengthening the work of the Church, and for evangelising the population of the metropolis. To this fund the Prince of Wales subscribed £1,000, and so successful has been the appeal that nearly £800,000 has been raised during the last twenty-four years. Most of this money has been expended upon grants in aid of 150 permanent churches, 139 of which are parochial, with separate districts and endowments, ministering to a population of nearly a million souls. Some 250 clergy, in addition to many lay agents, scripture-readers, and mission women, have been constantly engaged, owing to the assistance afforded by the fund.

It is further noteworthy that 132 of these churches have now permanent revenues, amounting in the aggregate to upwards of £46,000 per annum. Still much remains to be done. Some of the clergy in these new parishes, most of which are very poor, are heavily burdened in their efforts to maintain the existing organisations. The population of the diocese of London, though it has been reduced considerably in area since the inauguration of the fund, receives an increase of nearly 40,000 a year, and the moral, social, and spiritual care of this vast number is a matter of vital importance, and entails a work of great magnitude, which needs, and should continuously receive, year by year more and more support from the wealthy. No nobler effort can be conceived than that made by this fund to spread amongst the poorest classes of London the instruction and consolation of the Gospel.

In his letter to the churches, the Bishop states that the income of the Bishop of London's Fund, which had dropped in 1887 to less than £16,000, has risen again in 1888 to £23,000, at which figure it stood in 1886. The fluctuation is thus proved to be temporary, and it may be hoped will not recur. But even so, the fund is far from sufficient to supply the need, especially now that the Ecclesiastical Commissioners are no longer able, as heretofore, to endow churches as fast as they can be built. The Bishop is, therefore, obliged to press upon all Christians in his diocese, and particularly on all Churchmen, the duty of making earnest efforts to put the fund into a position to fulfil the purpose for which it was originally begun, and to provide in some reasonable degree for the spiritual care of the perpetually increasing population.

They had not only to help and stimulate the building of churches and formation of new parishes, but had to aid in the provision of clergy. There were large parishes with populations of ten or twelve thousand or more, with a total endowment of £200 a year for the clergyman. There were new parishes with no endowment at present, and no hope of any for some years to come, unless the fund can help to provide it. It was sad to witness, as the Bishop is compelled to do, the painful struggles of many of the clergy to do work which it is far beyond their real power to do; their severe pecuniary anxieties caused by their being driven to spend on their parishes what they ought to spend on themselves and their families; their failure in health caused by incessant labour and incessant worry.

The Ecclesiastical Commissioners have undertaken to help, though they cannot now do all. They will receive under special rules contributions towards the endowment either of incumbencies or of curacies, and will add a similar amount, and pay annually 3 per cent. on the whole, which practically comes to their paying 6 per cent. on the contributions paid in. And if the Bishop of London's Fund were enabled to aid these contributions by grants, much might soon be done to provide those clergy who are overburdened, and in danger of breaking down, with the needful aid for doing their work.

The Bishop, therefore, earnestly appeals for help in what so deeply concerns the spiritual welfare of our fellow-citizens, the life of the Church, and the morality and well-being of the whole community. Contributions may be sent to the Bishop, Fulham Palace, S.W.

